# Echocardiographic Changes Related to Pulmonary Hypertension in Preweaned Dairy Calves With Bronchopneumonia: A Case–Control Study in Commercial Dairy Farms

**DOI:** 10.1111/jvim.70020

**Published:** 2025-02-17

**Authors:** Sara Ghilardi, Giulia Sala, Chiara Locatelli, Davide Pravettoni, Mara Bagardi, Antonio Boccardo

**Affiliations:** ^1^ Dipartimento di Medicina Veterinaria e Scienze Animali (DIVAS) Università Degli Studi di Milano Lodi Italy; ^2^ Dipartimento di Scienze Veterinarie Università Degli Studi di Pisa San Piero a Grado Italy

**Keywords:** calves, echocardiography, respiratory disease, thoracic ultrasonography

## Abstract

**Background:**

Bronchopneumonia (BP) can cause pulmonary hypertension (PH) and secondary cardiovascular changes.

**Objectives:**

The aim of this study was to describe PH–related transthoracic Doppler echocardiography (TTE) changes in preweaned dairy calves with BP diagnosed by thoracic ultrasonography (TUS).

**Animals:**

One hundred and sixty‐four calves were selected from 11 commercial dairy farms.

**Methods:**

This is a case–control study. The enrolled calves were grouped according to TUS results into either the control group (with normally aerated lungs) or the BP group (calves with lobar BP). Both groups were then subjected to TTE.

**Results:**

Three echocardiographic variables were statistically different between the two groups, which included 104 healthy calves and 60 diagnosed with BP. The internal end‐systolic (LVIDs) and end‐diastolic diameters of the left ventricle (LVIDd) were significantly (*p* = 0.033, 0.034, respectively) lower in BP‐affected calves (mean ± standard deviation [SD]: LVIDs, 29.65 ± 3.94 mm in healthy calves vs. 28.21 ± 4.44 mm in BP‐affected calves; LVIDd, 49.83 ± 4.7 mm in healthy calves vs. 48.11 ± 5.4 in BP‐affected calves). The pulmonary artery internal diameter in end‐diastole (PAdia) was significantly larger (*p* = 0.017) in BP‐affected calves (16.81 ± 2.68 mm) than in healthy calves (15.75 ± 2.67 mm).

**Conclusions and Clinical Importance:**

The observed differences in the affected calves were within the normal reference ranges and exhibited clinical relevance. The lack of evident cardiac disturbances indicates that the BP diagnosis in our study sample was made without relevant cardiac alterations, highlighting the potential of TUS's early diagnostic capabilities during BP episodes.

**Abbreviations**
Ao: aorta/aortic annulus diameter
BP: bronchopneumonia
CV: coefficient of variation
ICS: intercostal spaces
IQR: interquartile range
IVS: interventricular septum
LVIDd: left ventricular internal diameter in diastole
LVIDs: left ventricular internal diameter in systole
mPAP: mean pulmonary artery pressure
PA: pulmonary artery / pulmonary artery annulus diameter
PA/Ao: pulmonary artery annulus‐to‐aortic annulus ratio
PA AT: pulmonary artery acceleration time
PA AT/ET: pulmonary artery acceleration‐to‐ejection time ratio
PAD: pulmonary artery distensibility
PAdia: pulmonary artery internal diameter in diastole
PA ET: pulmonary artery ejection time
PAP: pulmonary artery pressure
PAsys: pulmonary artery internal diameter in systole
PH: pulmonary hypertension
PR: pulmonary regurgitation
PRVmax: pulmonary regurgitation peak velocity
RA: right atrium
RAA: right atrium area
RV: right ventricle
RVH: right ventricular hypertrophy
RVOT: right ventricular outflow tract
RVW: right ventricular free wall
SD: standard deviation
sPAP: systolic pulmonary artery pressure
TAPSE: tricuspid annular plane systolic excursion
TR: tricuspid regurgitation‐derived maximal pressure gradient
TRVmax: tricuspid regurgitation peak velocity
TTE: transthoracic echocardiography
TUS: thoracic ultrasonography

## 1 Introduction

Bronchopneumonia (BP) is a relevant welfare concern in the cattle industry. In dairy calves, it leads to discomfort [1], reduces average daily gain [2], and increases culling and case fatality [3]. Furthermore, it is one of the most extensive diseases in cattle, contributing to the use of antimicrobials [4].

Thoracic ultrasonography (TUS) is considered a practical and accessible confirmatory test for the in vivo diagnosis of BP, with higher diagnostic performance than BP‐related clinical signs [5]. It shows an apparent ability to anticipate clinical manifestations [6, 7] that were frequently primarily observed in severe lung disorders [2, 8] or appeared without a corresponding lung consolidation [8, 9]. Evidence also indicates the long‐term effects of highlighted ultrasound lesions [2, 6]. However, the systemic impacts of the abnormalities accompanying ultrasound‐highlighted lesions on a calf's health remain unexplored.

In response to disease conditions, the lung activates an essential homeostatic mechanism to sustain normal oxygenation levels. This process constricts blood vessels in damaged areas and redirects blood flow away from poorly oxygenated regions [10]. Pulmonary vasoconstriction causes an increase in vascular resistance that can lead to pulmonary hypertension (PH), which, in humans, is considered as a mean pulmonary artery (PA) pressure (mPAP) > 20 mmHg at rest [11]. PA pressure in cattle has been primarily studied in research on respiratory compromise due to exposure to high altitudes [12]. Other studies have focused on transient pulmonary vascular remodeling related to adaptation to extrauterine life [13], after intratracheal inoculation of *Mannheimia haemolytica* [14], or as animal models evaluating obesity‐ and hypoxia‐associated PH [15, 16]. All studies on cattle have measured PAP using right heart catheterization, which is the definitive diagnostic tool for assessing this condition [17]. However, this invasive method is impractical and requires special equipment. To address this issue, in human and small animal medicine, the diagnostic accuracy of transthoracic Doppler echocardiography (TTE) has been studied as a noninvasive diagnostic tool that can assess the probability of PH based on cardiac modifications that occur due to pulmonary hemodynamic changes. Although TTE cannot replace right heart catheterization for accurate measurement of PAP, the ultrasonographic method has shown clinical value in analyzing cardiovascular changes secondary to PH [18, 19]. In these conditions, the typical findings during a TTE examination may include the following: enlargement of the pulmonary artery annulus diameter (PA; end‐diastolic pulmonary artery annulus‐to‐aortic annulus ratio [PA/Ao] > 1), reduction of the PA distensibility index (PAD), right ventricular hypertrophy (RVH), increased right atrium (RA) end‐systolic area (RAA), presence of systolic flattening or paradoxical motion of the interventricular septum (IVS), and high‐velocity tricuspid (TR) or pulmonary regurgitation (PR) jets [17, 20]. The diagnostic accuracy of these cardiac changes for diagnosing PH, assessed using right heart catheterization as the gold standard, has yet to be verified in species other than humans and dogs. However, TTE is commonly used to evaluate alterations that could be determined from PH in many animal species, such as pigs, sheep, murine models, and horses [20, 21, 22, 23], and has been widely used to estimate PH secondary to respiratory diseases [17, 24, 25].

Although PH has already been recognized in cattle and dogs with a high case fatality [12, 26], no previous study has investigated echocardiographic PH–related changes in preweaned dairy calves with naturally occurring BP diagnosed with TUS. Exploring the relationship between lung consolidations and the presence/severity of TTE signs related to PH could increase TUS' diagnostic value in assessing animals' physiological status. It also provides new insights into the cardiopulmonary changes, furthering our comprehension of how the disease impacts the health of preweaned dairy calves. Hence, the objective of this study was to delineate the TTE findings related to PH in a group of preweaned dairy calves with TUS–identified lung consolidations compared to a group without lung lesions.

We hypothesized that calves with lung consolidations would exhibit echocardiographic changes indicative of PH compared to those without.

## 2 Materials and Methods

### 2.1 Study Design and Ethics Statement

We conducted a case–control study adhering to the strengthening of reporting observational studies in epidemiology (STROBE) guidelines, utilizing a convenience sample of dairy calves from February to May 2024. We chose a case–control design because there was no information about PH–related TTE changes in preweaned dairy calves with TUS–highlighted lung lesions. Accordingly, we intentionally planned to emphasize differences between affected and unaffected calves to define the possible evidence for an association between TUS–detected lung lesions and TTE findings associated with PH.

This study was approved by the University of Milan's institutional animal welfare organization (approval number 54_2024).

### 2.2 Origin of the Calves

The selection of dairy farms focused on those who requested our diagnostic intervention for the presence of cough in the previous 7–10 days in preweaned calves housed in multiple pens with automatic feeders. The enrolled farms were located within a maximum driving distance of 60 min from the Clinic for Ruminants and Swine of the Department of Veterinary Medicine and Animal Science, University of Milan. Calves were housed individually for up to 15–20 days and fed with a milk replacer before being led into multiple pens (20–25 calves per pen) with an automated calf feeder for preweaning calves, where they remained for up to 75–95 days.

### 2.3 Eligibility Criteria

In enrolled herds, all Holstein‐Friesian preweaned calves housed in multiple pens were considered eligible for the study unless they had lameness, cachexia, diarrhea, or umbilical disease, or had received antimicrobial treatments within the preceding 15 days, which were established as exclusion criteria. We considered only female heifers eligible for the study. All calves in a preweaning pen on the enrolled farms were examined. In larger herds, when there was more than one pen to choose from, we selected the most convenient one for the study. Throughout the study, calves were initially evaluated on each farm using the same one‐gate reverse flow design, with cases and controls selected from a unified source of study samples [27].

In each enrolled pen, two assistants captured calves to undergo TUS. One of the principal authors (G.S.) examined each calf before TUS to identify any clinical signs that would lead to exclusion. In addition, the enrolled calves were evaluated for BP‐related clinical signs using the California (CALIF) respiratory scoring system by the same author and categorized as CALIF‐negative or CALIF‐positive if the score was < 5 or ≥ 5 [28], respectively.

### 2.4 Sampling Method and Sample Size Calculation

In our previous study, which was conducted with identical selection criteria in the same geographical area, the expected prevalence of severe lung lesions was approximately 40% [29]. We planned to select the first 15 eligible calves from each enrolled pen for TTE, aiming to include approximately 5 cases and 10 controls. By incorporating an unequal ratio of cases and controls (approximately 1:2), we sought to strengthen our case–control approach [30].

The sample size for this unmatched case–control study was determined using a *t*‐test analysis for the difference between two independent means to define the minimum sample size of calves needed to detect differences in TTE–related signs of PH between healthy and BP‐affected calves (G‐power v.3.1, Heinrich‐Heine‐Universität, Düsseldorf, Germany). An effect size of 0.5, *α* error of 5% (Type I), a confidence interval of 95%, and a test power of 80% were applied, resulting in a minimum sample size of 144 calves (48 cases and 96 controls). An effect size of 0.5 was selected based on existing literature regarding physiological echocardiographic measurements in calves and dogs with PH. The decision to use dogs as the reference species for assessing PH–related TTE abnormalities was made due to a lack of data on bovine species. The echocardiographic variable considered for the sample size calculation was the PA/Ao ratio. In dairy cows, the physiological median PA/Ao value was 0.86 ± 0.09 [31], while PH is suspected in dogs when the PA/Ao exceeds 1 [17]. Using this information, the determined effect size was 0.5.

### 2.5 Rationale for Choosing Cases and Controls

The results of TUS were utilized to select cases and controls for TTE, which was performed on the same day as TUS. Bilateral TUS (ICS 10‐1 on the right and ICS 10‐2 on the left) was performed based on the ventral landmarks described in previous studies [32] by the last author. The TUS was conducted using a portable unit (Draminski Blue, Draminski Ultrasound Scanners, Sząbruk, Poland) with a 7.5 MHz rectal transducer set to a depth of 8 cm. The thorax was not shaved, and 70% isopropyl alcohol was applied to the hair. TUS was categorized on a scale of 0–5 points using the lobe subdivision described in a previously published paper [33]. Calves with a TUS score ≥ 3 (lobar BP) were classified as positive cases; healthy calves with a TUS score of 0 or 1 (normal aerated lung parenchyma or presence of comet‐tail artifacts without consolidation) were considered controls. Calves with lobular pneumonia (TUS score of 2) did not undergo TTE. This selection criterion was implemented to ensure a clear distinction between healthy calves and those affected by the specific lung lesions associated with BP in dairy calves.

After choosing the cases and controls, the study design was changed to a two‐gate design, and the calves were moved to an area near the preweaning pen, where the workstation for performing TTE was set up.

### 2.6 TTE

A postdoctoral researcher with expertise in echocardiography (S.G.), who was blind to the TUS findings, conducted TTE using a MyLab Omega Vet ultrasound machine (Esaote S.p.A., Genova, Italy) equipped with a phased array probe (1 MHz). Throughout the procedure, the calves were standing and restrained with a halter. Before the TTE, the same author conducted cardiac auscultation, registering the absence or presence of a heart murmur. The detection of a cardiac murmur was not considered an exclusion criterion for this study as TTE was subsequently performed; calves affected by congenital cardiac diseases or valvular alterations attributable to endocarditis (such as valvular thickening, vegetative lesions associated with leaflets prolapse/flail, or valvular regurgitation) were excluded from the study. For each enrolled calf, the area from the third to the fifth intercostal space was clipped on the right and left side of the thorax in the cardiac region, and the skin was cleaned with 70% isopropyl alcohol. Echocardiograms were performed without continuous electrocardiographic monitoring, and video clips including at least three cardiac cycles were obtained to make offline measurements. For each echocardiographic variable, the recorded value was determined from the average of the three cardiac cycles. Following the reported guidelines for TTE in calves, a long‐axis four‐chamber view, a long‐axis five‐chamber view, a right parasternal cranial long‐axis view of the right ventricular outflow tract (RVOT), and the short‐axis views (at the level of the papillary muscles, the base of the heart, and the PA bifurcation) were obtained on the right side of the thorax [34]. An apical four‐chamber view optimized for the right heart was obtained on the left side of the thorax [35].

Echocardiographic measurements were obtained offline at the end of the study using the tools available in the ultrasound machine system by the same operator who performed the TTE. The detection and description (mild, moderate, and severe) [36] of TR through color Doppler and the quantification of its peak velocity (TRVmax) through continuous‐wave Doppler were performed on the long‐axis four‐chamber view or on the apical four‐chamber view optimized for the right heart (the view that provided the best alignment of the ultrasound beam with the regurgitant jet was chosen). The pressure gradient between the RV and the RA was derived by applying the simplified Bernoulli equation (PG = 4 × velocity [2]) to the TRVmax (TRPGmax) for the estimation of systolic PAP (sPAP) [17]. In the same way, the PR velocity (PRVmax) was used, when present and measurable, to estimate mPAP at the early diastolic peak [17]; its detection and description (mild, moderate, and severe) [36] were assessed on the right parasternal cranial long‐axis view of the RVOT.

Measurements from 2D images were obtained using the inner edge‐to‐inner edge method and included the end‐diastolic PA diameter and the end‐diastolic aortic (Ao) diameter [37]. End diastole was identified as the last frame before opening the semilunar valves. Measurements were made on the right parasternal cranial long‐axis view of the RVOT and the right parasternal long‐axis five‐chamber view [37]. Systolic IVS flatting was subjectively assessed on the short‐axis view at the level of the papillary muscles [17]. RVH was subjectively assessed on the right parasternal long‐axis four‐chamber view by evaluating the RV free wall (RVW): when RVW was more than half the size of the left ventricular free wall, the RV was considered hypertrophic [38]. Using the same echocardiographic view, RV dilation was subjectively assessed by comparing the right ventricular and the left ventricular internal size: when the right ventricular chamber size was more than a third of the left ventricular size, RV dilation was confirmed [38]. The RA dimension was evaluated on the right parasternal long‐axis four‐chamber view by measuring RAA as previously reported in dogs [39]; furthermore, RA enlargement was also subjectively assessed by the operator [18].

**FIGURE 1:**
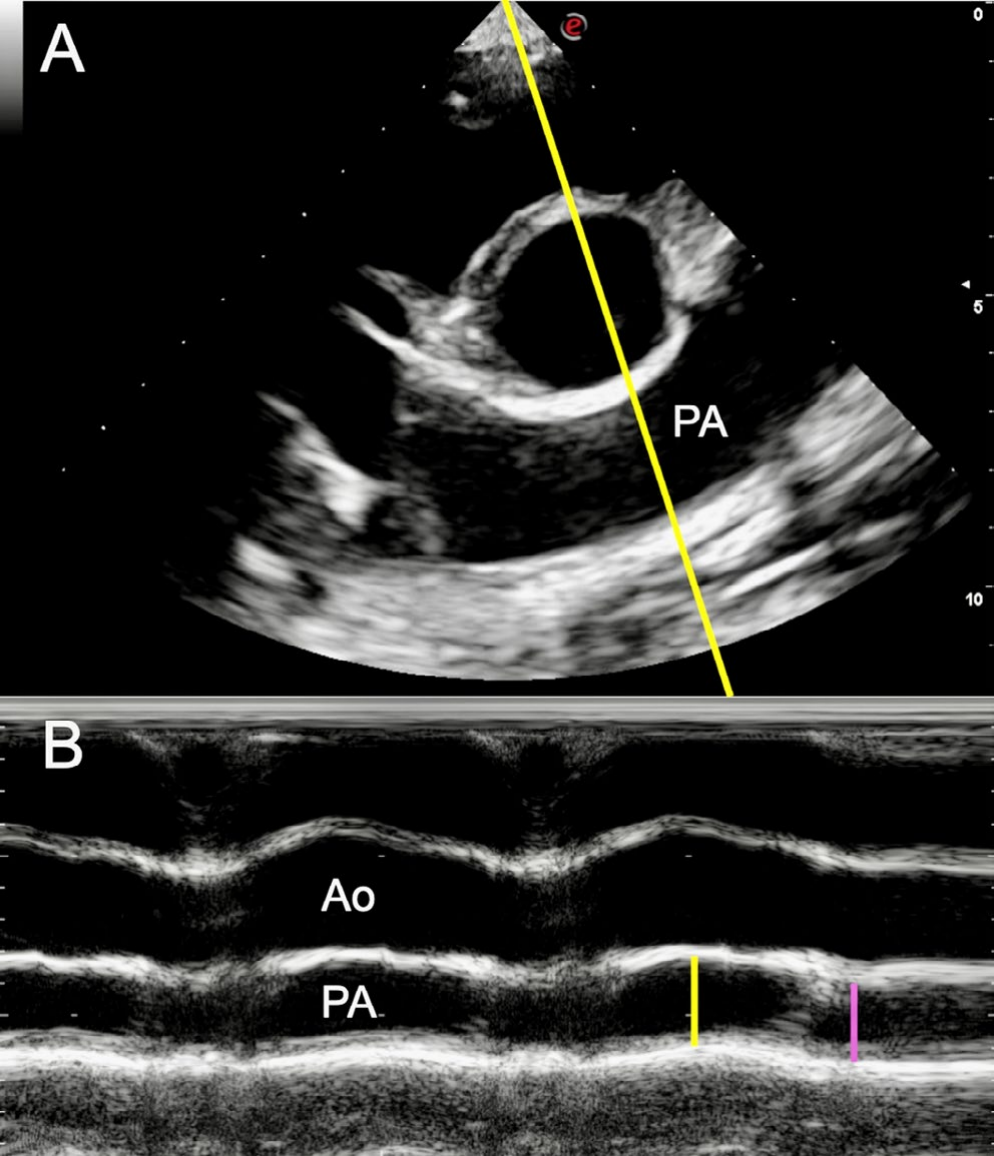
(A) Short‐axis right parasternal view of the cardiac base optimized to highlight the pulmonary artery bifurcation in a 66‐day‐old dairy calf enrolled in this study. The M‐mode imaging cursor (yellow line) was directed perpendicular to the longitudinal axis of the pulmonary artery (PA). (B) M‐mode view showing the aorta (Ao) and the pulmonary artery (PA) of the same calf. The yellow line traces the PA systolic diameter (PAsys) measurement; the purple line traces the PA diastolic diameter (PAdia), following the leading edge‐to‐leading edge method.

Measurements from M‐mode images included the internal end‐systolic and end‐diastolic diameters of the left ventricle (LVIDs and LVIDd, respectively), the tricuspid annular plane systolic excursion (TAPSE), and the PA internal diameter in end‐diastole (PAdia) and peak systole (PAsys). LVIDs and LVIDd were obtained from a 2D–directed image of the right short‐axis view at the level of the papillary muscles using the leading edge‐to‐leading edge method [37]; considering the absence of the electrocardiographic monitoring, the biggest (LVIDd) and the shortest (LVIDs) distance between the interventricular septum and the left ventricular free wall were considered. TAPSE was obtained on the apical four‐chamber view optimized for the right heart and measured as reported in dogs [40]. Finally, PA internal diameters were measured using a 2D–guided image of the right short‐axis view at the level of the base of the heart, optimized for the PA bifurcation, using the inner edge‐to‐inner edge method. M‐mode was performed by placing the cursor perpendicularly to the longitudinal axis of the pulmonary artery: PA systolic (PAsys) and diastolic (PAdia) diameters were subsequently obtained at the maximum and minimum size of the vessel, respectively (Figure 1).

Measurements of PA flow were obtained using the pulsed‐wave Doppler from the right parasternal cranial long‐axis view of the RVOT, placing the sample volume (2 mm) at the opening of the valvular leaflets. Acceleration time (AT) was measured from the onset of the flow profile to its peak velocity; ejection time (ET) was measured from the onset to the end of the flow profile [41].

The calculated measurements included PA/Ao, PAD index (PAD = [(PAsys − PAdia)/PAsys] × 100) [42], and the ratio between PA AT to ET (PA AT/ET) [41].

Five calves were randomly selected to assess intra‐ and inter‐operator variability. Intra‐operator agreement was determined by having the same operator (S.G.) perform all the TTE measurements offline on the five selected calves on three different days, 1–2 weeks apart, starting from at least 7 days after TTE was performed on the farm. Inter‐operator agreement was assessed by having three authors with expertise in TTE (S.G., M.B., and C.L.) perform the TTE measurements offline on the same five selected calves. Operators were blind to each other's measurements and their previous measurements. The choice of images and frames to obtain TTE measurements was at the discretion of each operator.

### 2.7 Statistical Analysis

Statistical analyses were performed with IBM SPSS Statistics v.29.0.2.0 (IBM Corp., Armonk, NY). The distribution of data was checked using the Shapiro–Wilk test. Continuous variables that were normally distributed were reported as average ± standard deviation (SD), whereas variables that were not normally distributed were reported as median and interquartile range (IQR) from the 25th to the 75th percentile. Categorical variables were expressed as frequencies and percentages.

Differences between continuous variables were analyzed in the healthy calves (TUS scores of 0 and 1) versus BP‐affected calves (TUS scores of 3, 4, and 5) using the *T*‐test or Mann–Whitney *U*‐test, chosen according to data distribution. The chi‐squared test evaluated differences in categorical variables between healthy calves (TUS scores of 0 and 1) versus BP‐affected calves (TUS scores of 3, 4, and 5). Statistical significance was set for a *p* value < 0.05. Previous literature reported that TTE measures are related to age [43]; therefore, age distribution between groups was tested using the *T*‐test, and the correlation between age and other TTE measures variables was tested with the Pearson correlation test. The Pearson correlation coefficient (*r*) was interpreted as follows: < 0.10 as negligible correlation, 0.10–0.39 as weak correlation, 0.40–0.69 as moderate correlation, 0.70–0.89 as strong correlation, and 0.90–1.00 as robust correlation [44].

In addition, differences between continuous variables in CALIF‐negative calves (score < 5) and CALIF‐positive calves (score ≥ 5) were analyzed using either the *T*‐test or Mann–Whitney *U*‐test based on data distribution. Differences in categorical variables were evaluated with the chi‐squared test. A Kruskal–Wallis test or one‐way ANOVA was used to assess differences in continuous echocardiographic variables across calves categorized by a combination of CALIF and TUS scores. Details of these analyses are reported in Data S2.

Intra‐ and inter‐operator agreement for TTE measurements was assessed through the coefficient of variation (CV) using the following formula: CV (%) = (standard deviation/mean) × 100 in five randomly selected calves. Calves were randomly chosen with an Android application (Randomizer, Darshan Institute of Engineering and Technology, Rajkot, India) using the total list of calves (numbered from 1 to 164) that underwent TTE. Three groupings were created (Calves 1–54, 55–110, and 111–164), and five numbers were randomly selected from these three sets. Echocardiographic measurements were obtained offline by each operator on three different days, 1–2 weeks apart. The three measurements on each calf were used to obtain CV for every echocardiographic variable, and then the average of the CV in the five selected animals was reported.

The degree of variability was considered as previously reported in the literature: CV < 5%, excellent repeatability; CV 5%–15%, good repeatability; CV 15%–25%, moderate repeatability; CV > 25%, poor repeatability [45].

## 3 Results

During our study, we visited 11 dairy farms and conducted TUS on 182 eligible calves. Seventeen calves were excluded from the study because of a TUS score of 2. Additionally, one enrolled calf was excluded after TTE due to a diagnosis of *cor triatriatum sinister*. The final sample consisted of 164 female Holstein‐Friesian calves aged 18–96 (56.8 ± 17.2 days). Of these calves, 104 were deemed healthy (62 had TUS scores of 0, and 42 had scores of 1), while 60 were diagnosed with BP (28 had TUS scores of 3, 24 had TUS scores of 4, and 8 had TUS scores of 5). The detailed clinical data, age, farm of origin, TUS, and TTE findings for each calf included in the final analysis are provided in Data S1. The distribution of healthy and BP‐affected calves across the farms was as follows:

- Farms 1 and 2: 10 healthy calves and 5 BP‐affected calves
- Farm 3: 8 healthy calves and 7 BP‐affected calves
- Farm 4: 7 healthy calves and 8 BP‐affected calves
- Farms 5, 9, and 11: 11 healthy calves and 4 BP‐affected calves
- Farm 6: 9 healthy calves and 6 BP‐affected calves
- Farm 7: 7 healthy calves and 8 BP‐affected calves
- Farm 8: 12 healthy calves and 3 BP‐affected calves

Owing to adverse weather conditions that affected the continuation of the study on Farm 10, we could only enroll 14 calves (9 healthy and 5 with BP) on this farm.

Cardiac auscultation was conducted on all 164 TTE–enrolled calves, revealing two cases of heart murmurs: a left systolic murmur (intensity I/VI) and a left diastolic murmur (intensity II/VI). No cardiac abnormalities were observed during the TTE; consequently, these two calves were enrolled for the final analyses. TR was detected in 25 calves, but the operator consistently deemed it mild. Only one calf (0.61%) had a measurable TR velocity (TRVmax: 1.89 m/s), which did not suggest high sPAP; in the remaining 24 (14.63%) calves, regurgitant jets were too mild for their velocity to be detected through continuous‐wave Doppler. PR was identified in 118 calves and was consistently assessed as mild. Peak PR velocity was only measured in four cases (2.44%), never indicating mPAP greater than 20 mmHg (PRVmax ranged from 1.43 to 2.2 m/s); as reported for TR in the majority of cases (69.51%), PR was too mild for the assessment of its peak velocity. Challenges were encountered in consistently aligning the probe on the left side of the thorax to obtain an optimally oriented apical four‐chamber view for the right heart. As a result, TAPSE was deemed an unreliable echocardiographic measurement and was consequently excluded from the analysis.

Only PAD, PA AT/ET, and RAA were not normally distributed. The average or median values of the TTE variables and their statistical significance are presented in Table 1, divided between healthy and BP‐affected calves. The results highlight a statistically significant difference between healthy and BP‐affected calves for LVIDd and LVIDs, with lower values observed in BP‐affected calves (LVIDd: healthy 49.83 ± 4.70 mm; BP 48.11 ± 5.40 mm; *p* = 0.034; LVIDs: healthy 29.65 ± 3.94 mm; BP 28.21 ± 4.44 mm; *p* = 0.033). Additionally, PAdia significantly differed between healthy and affected calves, with a higher value in BP‐affected calves (healthy 15.75 ± 2.67 mm; BP 16.81 ± 2.68 mm; *p* = 0.017).

**TABLE 1:** Differences in transthoracic Doppler echocardiography measurements among 164 preweaned dairy calves; 60 were diagnosed with lobar bronchopneumonia (BP) and 104 without BP via thoracic ultrasonography^f^.

Variables	Healthy calves (*n* = 104)		BP calves (*n* = 60)		*p*
	Mean ± SD	Min–max	Mean ± SD	Min—max	
	Median^a^ (25th–75th IQR)^b^		Median^a^ (25th–75th IQR)^b^		
Linear and volumetric measurements					
LVIDd (mm)	49.83 ± 4.7^#^	39.5–62.7	48.11 ± 5.4^#^	36.8–67.1	0.034^c^
LVIDs (mm)	29.65 ± 3.94^#^	22–40.2	28.21 ± 4.44^#^	18.7–39.1	0.033^c^
PA (mm)	26.94 ± 2.78	21.1–35.1	26.65 ± 3.16	21.1–35.4	0.545^c^
Ao (mm)	28.55 ± 2.6	23.7–36.4	28.5 ± 2.6	22.9–36.2	0.902^c^
PA/Ao	0.945 ± 0.087	0.75–1.25	0.936 ± 0.082	0.734–1.164	0.514^c^
PAD (%)	31.53^a^ (27–34.61)^b^	16.14–57.99	29.88^a^ (24.6–34.68)^b^	14.09–42.8	0.167^d^
PAsys (mm)	23.08 ± 3.12	16.2–30.6	23.87 ± 2.93	16.9–29.6	0.117^c^
PAdia (mm)	15.75 ± 2.67^#^	9.2–21.4	16.81 ± 2.68^#^	11.0–22.9	0.017^c^
PA AT (ms)	121.78 ± 19.53	78–186	125 ± 18.79	86–166	0.263^c^
PA ET (ms)	293.63 ± 44.34	166–417	307.04 ± 48.08	204–455	0.077^c^
PA AT/ET	0.41^a^ (0.38–0.44)^b^	0.30–0.66	0.40^a^ (0.38–0.43)^b^	0.29–0.53	0.399^d^
RAA (cm^2^)	13.23^a^ (11.58–14.98)^b^	7.08–22.68	12.5^a^ (11.11–14.13)^b^	8.86–22.17	0.152^d^
Visual/subjective measurements					
Flattening IVS	Negative cases: 100%		Negative cases: 100%		^e^
RVH	Negative cases: 71.4%		Negative cases: 71.2%		0.974^e^
	Positive cases: 28.6%		Positive cases: 28.8%		
PR presence	Negative cases: 26.7%		Negative cases: 22.8%		0.586^e^
	Positive cases: 73.3%		Positive cases: 77.2%		
RA enlargement	Negative cases: 72.1%		Negative cases: 77.6%		0.446^e^
	Positive cases: 27.9%		Positive cases: 22.4%		
TR presence	Negative cases: 83.6%		Negative cases: 86.4%		0.635^e^
	Positive cases: 16.4%		Positive cases: 13.6%		

*Note:* Continuous variables with normal distribution are presented as mean ± standard deviation (SD), whereas non‐normally distributed variables are presented as median and IQR from the 25th to the 75th percentile. Continuous variables between healthy and BP‐affected calves were assessed using the *T*‐test or Mann–Whitney *U*‐test based on data distribution. The chi‐squared test was utilized for categorical variables. Statistical significance was established at *p* < 0.05.

Abbreviations: Ao, aortic annulus diameter; IVS, interventricular septum; LVIDd, left ventricular internal diameter in diastole; LVIDs, left ventricular internal diameter in systole; PA, pulmonary artery annulus diameter; PAD, pulmonary artery distensibility index; PAdia, pulmonary artery diameter in diastole; PAsys, pulmonary artery diameter in systole; PA AT, pulmonary artery acceleration time; PA AT/ET, pulmonary artery acceleration‐to‐ejection time ratio; PA ET, pulmonary artery ejection time; PA/Ao, pulmonary artery annulus‐to‐aortic annulus ratio; PR, pulmonic regurgitation; RA, right atrium; RAA, right atrium area; RVH, right ventricular hypertrophy (eccentric or concentric); TR, tricuspid regurgitation.

^a^ Median.

^b^ Interquartile range (IQR) from the 25th to the 75th percentile.

^c^ *T*‐test.

^d^ Mann–Whitney *U*‐test.

^e^ Chi‐square of Pearson.

^f^ Thoracic ultrasonography was graded on a scale of 0–5 points using the lobe subdivision outlined in a previously published paper (Ollivett et al. [33]). Calves with a TUS score of 3 or higher were classified as positive cases affected by BP, whereas calves with a TUS score of 0 or 1 were considered negative controls. This study did not include calves with lobular BP (TUS Score 2).

^#^ Statistically different (*p* < 0.05).

The PAD index, PA/Ao, and RAA were not statistically different between healthy and BP‐affected calves. Similarly, subjective measures of RVH and RA enlargement did not differ between healthy and affected calves, and IVS flattening was not detected in any of the calves enrolled for TTE.

Our study revealed a significant correlation between age and some echocardiographic variables. Age was found to be moderately correlated with LVIDd (*r* = 0.50), PA (*r* = 0.46), and Ao (*r* = 0.67) and weakly correlated with LVIDs (*r* = 0.32) and PAsys (*r* = 0.33). However, the age distribution between healthy calves (mean value 55.1 ± 16.8 days) and BP‐affected calves (mean value 59.7 ± 17.4) did not show a statistically significant difference (*p* = 0.099).

No statistically significant differences were observed for any TTE variables, whether using CALIF alone or in combination with TUS (details in Data S2).

Table 2 shows the mean variation coefficients (CVs) for intra‐ and inter‐operator agreement in TTE measurements. The findings indicate good to excellent repeatability for selected TTE measurements among different observers.

**TABLE 2:** Intra‐ and inter‐operator mean coefficients of variation (CV) were assessed for three B‐mode, four M‐mode, and two Doppler measurements obtained from five randomly selected calves out of the 164 that underwent TTE.

B‐mode	Mean CV (%)		M‐mode	Mean CV (%)		Doppler	Mean CV (%)	
	Intra‐operator	Inter‐operator		Intra‐operator	Inter‐operator		Intra‐operator	Inter‐operator
PA (mm)	3.08	6.66	LVIDd (mm)	1.81	2.93	PA AT (ms)	5.22	5.34
Ao (mm)	1.91	4.95	LVIDs (mm)	2.49	4.47	PA ET (ms)	6.66	8.31
RAA (cm^2^)	7.55	9.27	PAdia (mm)	3.02	6.99			
			PAsys (mm)	3.44	6.45			

*Note:* These measurements were derived from images captured by a single operator who performed TTE on all enrolled calves. Mean CV were classified as follows: a CV of less than 5% indicates excellent repeatability; a CV between 5% and 15% signifies good repeatability; a CV of 15%–25% reflects moderate repeatability; and a CV exceeding 25% denotes poor repeatability.

Abbreviations: Ao, aortic annulus diameter; LVIDd, left ventricular internal diameter in diastole; LVIDs, left ventricular internal diameter in systole; PA AT, pulmonary artery acceleration time; PA ET, pulmonary artery ejection time; PA, pulmonary artery annulus diameter; PAdia, pulmonary artery diameter in diastole; PAsys, pulmonary artery diameter in systole; RAA, right atrium area.

## 4 Discussion

In contrast to our initial hypothesis, this study indicates that BP detected by the TUS was characterized by disturbances associated with low effects on PH–related TTE findings in preweaned dairy calves. We identified statistically significant differences between healthy and affected calves in left ventricular end‐systolic internal diameters (LVIDd and LVIDs) and pulmonary artery diameter (PAdia). However, the anomalies observed in the affected calves did not substantially deviate from the reference ranges established for calves of the same age [31] and were numerically comparable to those of healthy calves. Furthermore, TR jets were infrequent compared to the numerically more conspicuous PR jets. However, the detection of neither jet significantly differed between healthy and diseased calves, and they also had a weak velocity to be detected by continuous‐wave Doppler. Analogously, PA/Ao and RAA showed no statistically significant differences between the group of healthy calves and those with lobar BP. These observations might support the hypothesis that PH–related echocardiographic changes in preweaned dairy calves with BP diagnosed by TUS were poor.

Transthoracic Doppler echocardiographic findings related to PH secondary to respiratory diseases in other species were commonly reported in obstructive/restrictive lung diseases [20, 24, 25, 46, 47]. The pathophysiological mechanisms contributing to the arteries anomalies in lung disease–related PH have yet to be fully understood [48]. Under these conditions, extensive lung tissue damage leads to inflammatory cytokine expression that could cause pulmonary artery remodeling. This remodeling involves changes in the endothelial cells, smooth muscle cells, and fibroblasts, contributing to intimal hypertrophy, endothelial dysfunction, and progressive fibrotic injury, leading to obliteration of pulmonary arteries and an overall increase in pulmonary blood pressure [49, 50]. Similarly, hypoxia‐related PH in the absence of lung disease, such as chronic exposure to high altitude that affects species not genetically predisposed to survive in these habitats, seems to be triggered by the low alveolar oxygen tension that enhances arterioles' resistance, resulting in contraction of vascular smooth muscle; nonetheless, prolonged exposure to low oxygen levels can lead to increased pulmonary blood pressure and the potential development of PH [51].

Two factors could explain our study sample's paucity of PH–related cardiovascular changes.

Infectious bovine BP is typically bilateral and cranioventrally distributed [52]. Conversely, lung lesions in the dorsocaudal portions are rare and almost always indicate poor prognosis [33]. Even under normal conditions, the cranioventral area of calves' lungs has less ventilation capacity than the dorsocentral areas [53]. It can be deduced that although some cranioventral areas were consolidated, calves diagnosed with BP in our study had no hemodynamically relevant alterations in pulmonary blood flow shunting, so no secondary cardiac alterations were present. Notably, most calves in our study sample exhibited only a single consolidated ventral‐cranial lung lobe (TUS Score 3). This observation underscores that this type of lesion remains too limited to alter pulmonary blood flow substantially. Furthermore, severe lung lesions were identified in only three calves in the dorsocaudal region of the lungs (refer to Table S1).

Another possible explanation for this phenomenon could be related to the acute lesions in our study sample. Echocardiographic alterations related to PH commonly correlate with chronic hypoxemia and pulmonary disease conditions [41, 54, 55]. Although more chronic cases may be observed in a high prevalence BP setting [5], and clinical signs often manifest much later than ultrasound lesions [2], it can be assumed that the cardiocirculatory anatomical alterations highlighted by TTE probably develop over a more extended period. However, these must be considered just as a hypothesis since right heart catheterization, the only diagnostic tool that could have confirmed the absence of a PAP increase, was not performed. Additionally, since we did not carry out a longitudinal study, we are uncertain about the true prevalence of chronic cases. Consequently, it is essential to recognize that our study sample may have overlooked some PH representatives due to the absence of right heart catheterization and the potential inclusion of calves with acute lung lesions. Nevertheless, it is worth noting that any overlooked alterations were not severe enough to induce cardiac alterations.

The results of our study can be extrapolated from other comparable studies on cattle. Recent studies reported that calves diagnosed with BP through TUS showed only moderate alterations in metabolic, arterial blood gases, and acid–base disorders [56]. These findings were also highlighted by others [57] who did not detect hypoxemia and hypercapnia after endoscopic inoculation of *Mannheimia haemolytica* . On the other hand, further studies showed that experimental infection with aggressive pathogens [58, 59], with a high dosage of inoculated bacteria [14], or life‐threatening conditions related to chronic BP in hospitalized calves [60] could impact blood oxygenation values and potentially increase PAP. The calves in this study were not subjected to arterial blood gas analysis. However, the lung lesions detected were not particularly severe enough to induce hypoxemia or generalized inflammatory conditions of lung tissue that would have led to considerable PH–related echocardiographic abnormalities.

Although the diagnosis of BP with TUS did not reveal substantial TTE changes, this result is nevertheless interesting. The findings of this case–control study indicate that under field conditions, TUS can provide diagnostic information for BP even in the absence of TTE signs of PH, especially in cases of acute onset. Compatible with recent literature, early treatment of non‐chronic lung lesions detected with TUS may improve recovery by identifying and treating infectious lesions before they cause functional damage [56, 61]. Future research must clarify whether chronic lung lesions or large lesions extending to the dorsocaudal portion are associated with more severe cardiopulmonary and functional changes. It is critical to determine if these changes are responsible for well‐documented production and growth consequences in dairy calves affected by this disease.

This study has some limitations. The investigation was carried out on a group of dairy farms selected for their convenience and known to have respiratory issues. Although these farms represent those in the Po Valley territory, there may have been a selection bias. We have mitigated this issue by employing an initial single‐gate reverse flow design for each enrolled farm, in which cases and controls were sampled from a single source study sample; this design includes cases from individuals with the same BP‐related pathogens exposure characteristics, allowing uninflated results to become more applicable to a broad context [27]. The observational approach of this study restricts our understanding of the temporal development of lung lesions within our study sample. A longitudinal TUS assessment of these lesions could have yielded different results, mainly if we had included participants with confirmed chronic lung conditions, who are more susceptible to exhibiting TTE signs associated with PH. Additionally, microbiological tests could not be conducted on these farms, preventing us from associating the TTE findings with specific pathogens encountered. TTE was performed without concurrent electrocardiographic monitoring, so conduction disturbances could not be detected. However, rhythm alterations were not identified during auscultation. Furthermore, previous studies were conducted without electrocardiographic monitoring [34, 43, 62, 63]. This could also be related to some practical difficulties in applying the electrodes under field conditions. Also, TTE is not the gold standard for diagnosing PH since the measurement of systolic, mean, and diastolic PAP can only be obtained through right heart catheterization. Although some authors describe right heart catheterization as a feasible technique in the field [10], the method poses difficulties on farms due to the necessity of specialized equipment and the inherent infection risks [64]. For this study, we adopted a globally accepted tool for estimating the probability of the disease in humans and many mammalian species, which, although it may have diagnostic limitations, allowed us to conduct the study.

In conclusion, TTE revealed no signs of alterations associated with PH in calves with lobar BP diagnosed by TUS. The absence of cardiac disturbances suggests that the diagnosis of BP occurred before considerable cardiac alterations could develop or that the detected lesions affected a small percentage of lung tissue, which was insufficient to cause noteworthy cardiac alterations.

## Disclosure

Authors declare no off‐label use of antimicrobials.

## Ethics Statement

This study was approved by the University of Milan's institutional animal welfare organization (approval number 54_2024). The authors declare that human ethics approval was not needed for this study.

## Conflicts of Interest

The authors declare no conflicts of interest.

## Supporting information


Data S1.

Data S2.
