# Supplementary material for: Echocardiographic Changes Related to Pulmonary Hypertension in Preweaned Dairy Calves With Bronchopneumonia: A Case–Control Study in Commercial Dairy Farms
Source: J Vet Intern Med. 2025 Feb 17;39(2):e70020. doi: 10.1111/jvim.70020 (PMC11831072; doi:10.1111/jvim.70020)
Supplement: Supplementary file 2 — Data S2. [file JVIM-39-e70020-s001.docx]

**Supplementary materials**

*Statistical analysis*

Differences between continuous variables were analyzed in CALIF-negative calves (CALIF score < 5) vs. CALIF-positive calves (CALIF score ≥ 5) using either the T-test or the Mann-Whitney U-test, depending on data distribution. Differences in categorical variables between CALIF-negative and CALIF-positive calves were evaluated using the chi-square test. Additionally, a Kruskal-Wallis test or one-way ANOVA, based on data distribution, was applied to highlight differences in continuous echocardiographic parameters across calves categorized using a combination of CALIF and TUS scores. The categorization was as follows: healthy (CALIF < 5 without consolidation on TUS), upper respiratory tract infection (CALIF ≥ 5 without consolidation on TUS), subclinical BP (CALIF < 5 with TUS > 3 cm), and clinical BP (CALIF ≥ 5 with TUS > 3 cm). The chi-square test was also employed to evaluate differences in categorical variables using the same categorization. Statistical significance was set at P < 0.05.

**Results**

Only PAD, PA AT/ET, and RAA were not normally distributed. The mean or median values of the TTE parameters and their statistical significance are presented in Table S1, categorized by CALIF-negative and CALIF-positive calves. Table S2 provides descriptive statistics for the categories of healthy, upper respiratory tract infection, subclinical BP, and clinical BP calves. No statistically significant differences were observed for any TTE parameters, whether using CALIF alone or in combination with TUS. Additionally, IVS flattening was not detected in any of the calves enrolled for TTE.

**Table S1**. Differences in transthoracic Doppler echocardiography measurements among 162 pre-weaned dairy calves (for two of the enrolled calves, CALIF data were missing); 127 were California Score (CALIF) negative, and 35 were CALIF positive*. Continuous variables with normal distribution are presented as mean ± standard deviation (SD), while non-normally distributed variables are presented as median and interquartile range (IQR) from the 25th to the 75th percentile. Continuous variables between CALIF positive and negative calves are assessed using the T-test or Mann-Whitney U-test based on data distribution. The chi-square test was utilized for categorical variables. Statistical significance was established at P <0.05.

| Parameters | CALIF negative calves (n. = 127) | | CALIF positive calves (n. = 35) | | P |
| --- | --- | --- | --- | --- | --- |
|  | *Mean ± SD*  *Median^a^ (25^th^ - 75^th^* IQR*)^b^* | *min - max* | *Mean ± SD*  *Median^a^ (25^th^ - 75^th^* IQR*)^b^* | *min – max* |  |
| Linear and volumetric measurements | | | | | |
| LVIDd (mm) | 49.47 ± 5.18 | 36.80 – 67.10 | 48.40 ±4.42 | 38.80 – 56.60 | 0.270^c^ |
| LVIDs (mm) | 29.20 ± 4.18 | 18.70 – 40.20 | 29.07 ± 4.17 | 22.30 – 39.10 | 0.878^c^ |
| PA (mm) | 26.90 ± 2.97 | 21.10 – 35.40 | 26.50 ± 2.79 | 21.10 – 31.00 | 0.484^c^ |
| Ao (mm) | 28.57 ±2.64 | 23.70 – 36.40 | 28.28 ± 2.49 | 22.90 – 33.60 | 0.559^c^ |
| PA/Ao | 0.94 ± 0.08 | 0.75 – 1.25 | 0.94 ±0.09 | 0.73 – 1.13 | 0.877^c^ |
| PAD (%) | 30.74 ^a^ (25.86 – 34.22)^b^ | 26.14 – 57.99 | 31.62 ^a^ (25.32 – 34.88)^b^ | 14.09 – 46.02 | 0.910^d^ |
| PAsys (mm) | 23.53 ± 3.08 | 16.20 – 30.60 | 22.87 ± 3.01 | 16.80 – 29.60 | 0.267^c^ |
| PAdia (mm) | 16.20 ± 2.70 | 9.20 – 21.70 | 16.00 ± 2.80 | 11.00 – 22.90 | 0.695^c^ |
| PA AT (ms) | 122.03 ±18.93 | 78.00 – 170.00 | 126.29 ± 20.94 | 86.00 – 186.00 | 0.253^c^ |
| PA ET (ms) | 297.39 ± 45.82 | 166.00 – 455.00 | 302.71 ± 47.79 | 204.00 – 417.00 | 0.548^c^ |
| PA AT/ET | 0.41^a^ (0.39 – 0.44)^b^ | 0.30 – 0.52 | 0.40 ^a^ (0.38 – 0.45)^b^ | 0.30 – 0.66 | 0.813^d^ |
| RAA (cm^2^) | 13.01 ^a^ (11.55 – 14.79)^b^ | 7.08 – 22.17 | 12.47 ^a^ (11.10 – 14.15)^b^ | 8.91 – 20.81 | 0.277^d^ |
| Visual/subjective measurements | | | | | |
| Flattening IVS | negative cases: 100% | | negative cases: 100% | | ^e^ |
| RVH | negative cases: 74,0 %  positive cases: 26.0 % | | negative cases: 62.9 %  positive cases: 37.1 % | | 0.195^e^ |
| PR presence | negative cases: 25.4 %  positive cases: 74.6 % | | negative cases: 23.5 %  positive cases: 76.5 % | | 0.823^e^ |
| RA enlargement | negative cases: 75.4 %  positive cases: 24.6 % | | negative cases: 70.6 %  positive cases: 29.4 % | | 0.569^e^ |
| TR presence | negative cases: 84.1 %  positive cases: 15.9% | | negative cases: 88.5 %  positive cases: 11.4 % | | 0.514^e^ |

^a^ median; ^b^ 25th and 75th percentile; ^c^ T-test; ^d^ Mann-Whitney U-test; ^e^ Chi-square of Pearson.

Ao, aortic annulus diameter; IVS, interventricular septum; LVIDd, left ventricular internal diameter in diastole; LVIDs, left ventricular internal diameter in systole; PA, pulmonary artery annulus diameter; PAD, pulmonary artery distensibility index; PAdia, pulmonary artery diameter in diastole; PAsys, pulmonary artery diameter in systole; PA AT, pulmonary artery acceleration time; PA AT/ET, pulmonary artery acceleration-to-ejection time ratio; PA ET, pulmonary artery ejection time; PA/Ao, pulmonary artery annulus-to-aortic annulus ratio; PR, pulmonic regurgitation; RA, right atrium; RAA, right atrium area; RVH, right ventricular hypertrophy (eccentric or concentric); TR, tricuspid regurgitation.

* The California scoring system evaluates six clinical criteria: nasal discharge, ocular discharge, rectal temperature, ear position, spontaneous coughing, and abnormal breathing. Each clinical sign is assigned a specific weight, reflecting its significance. The California score was deemed positive when the total score reached or exceeded 5.

**Table S2.** Differences in transthoracic Doppler echocardiography measurements among 162 pre-weaned dairy calves; 86 were healthy, 41 had an upper respiratory tract infection, 18 had subclinical bronchopneumonia (BP), and 17 had clinical BP*. Continuous variables with normal distribution are presented as mean ± standard deviation (SD), while non-normally distributed variables are presented as median and interquartile range (IQR) from the 25th to the 75th percentile. Continuous variables between the fourth classes of calves are assessed using the One-way ANOVA or Kruskal-Wallis based on data distribution. The chi-square test was utilized for categorical variables. Statistical significance was established at P <0.05.

|  | Healthy calves (n. = 86) |  |  | Upper respiratory tract  infection calves (n. = 41) |  |  | Subclinical BP calves (n. =18) |  |  | Clinical BP calves (n. = 17) |  | P |
| --- | --- | --- | --- | --- | --- | --- | --- | --- | --- | --- | --- | --- |
| Linear and volumetric measurements | | | | | | | | | | | | |
|  | *Median^a^ (25^th^ - 75^th^)^b^* | *min - max* |  | *Median^a^ (25^th^ - 75^th^)^b^* | *min - max* |  | *Median^a^ (25^th^ - 75^th^ )^b^* | *min - max* |  | *Median^a^ (25^th^ - 75^th^ )^b^* | *min - max* |  |
| LVIDd (mm) | 50.15 ± 4.68 | 39.5 – 62.70 |  | 48.03 ± 5.91 | 36.80 – 67.10 |  | 48.27 ± 4.78 | 40.70 – 56.60 |  | 48.55 ±4.15 | 38.80 – 55.00 | 0.104^c^ |
| LVIDs (mm) | 29.76 ± 3.99 | 22.00 – 40.20 |  | 28.01 ± 4.38 | 18.70 – 38.20 |  | 29.12 ± 3.91 | 22.30 – 35.20 |  | 29.02 ± 4.55 | 22.50 – 39.10 | 0.176^c^ |
| PA (mm) | 27.12 ±2.78 | 22.20 – 35.10 |  | 26.42 ± 3.32 | 21.10 – 35.40 |  | 25.98 ± 2.73 | 21.10 – 30.00 |  | 27.06 ± 2.83 | 21.50 – 31.00 | 0.357^c^ |
| Ao (mm) | 28.61 ±2.65 | 23.70 – 36.40 |  | 28.49± 2.65 | 24.40 – 36.20 |  | 28.16 ±2.44 | 24.20 – 32.70 |  | 28.41 ± 2.62 | 22.90 – 33.60 | 0.922^c^ |
| PA/Ao | 0.95 ± 0.09 | 0.75 – 1.25 |  | 0.93 ±0.08 | 0.77 – 1.16 |  | 0.93 ± 0.09 | 0.76 – 1.13 |  | 0.96 ±0.09 | 0.73 – 1.11 | 0.419^c^ |
| PAD (%) | 31.54 ^a^ (27.2 – 34.5)^b^ | 16.14 – 57.99 |  | 27.91^a^ (24.5 – 33.79)^b^ | 18.70 – 42.80 |  | 29.35 ^a^ (25.42 – 37.35)^b^ | 17.79 – 46.02 |  | 31.95 ^a^ (24.52 – 34.73)^b^ | 14.09 – 38.49 | 0.411^d^ |
| PAsys (mm) | 23.33 ± 3.15 | 16.20 – 30.60 |  | 23.95 ± 2.94 | 16.80 – 29.60 |  | 21.82 ± 2.08 | 16.20 – 27.20 |  | 23.92 ± 2.91 | 16.90 – 29.60 | 0.096^c^ |
| PAdia (mm) | 15.88 ± 2.73 | 9.10 – 21.40 |  | 16.87 ± 2.56 | 11.00 – 21.70 |  | 15.09 ± 2.45 | 11.00 – 19.60 |  | 16.91 ± 2.91 | 11.50 – 22.90 | 0.054^c^ |
| PA AT (ms) | 120.72 ± 19.10 | 78– 170 |  | 124.90 ± 18.48 | 88– 162 |  | 126.50 ±21.88 | 89– 186 |  | 126.06 ± 20.56 | 86– 166 | 0.469^c^ |
| PA ET (ms) | 293.20 ± 43.90 | 166– 384 |  | 306.51 ± 49.09 | 232 – 455 |  | 297.78 ± 47.83 | 216.00 – 417.00 |  | 307.94 ± 48.64 | 204.00 – 410.00 | 0.393^c^ |
| PA AT/ET | 0.41^a^ (0.39 – 0.44)^b^ | 0.33 – 0.52 |  | 0.41 ^a^ (0.39 – 0.44)^b^ | 0.30 – 0.49 |  | 0.42^a^ (0.39 – 0.45)^b^ | 0.30 – 0.66 |  | 0.39 ^a^ (0.37 – 0.44)^b^ | 0.36 – 0.53 | 0.759^d^ |
| RAA (cm^2^) | 13.3 ^a^ (11.8 – 15.03)^b^ | 7.08 – 21.93 |  | 12.34 ^a^ (11.1 – 14.08)^b^ | 8.86 – 22.17 |  | 12.03 ^a^ (11.04 – 14.18)^b^ | 8.91 – 17.05 |  | 12.59 ^a^ (11.04 – 14.28)^b^ | 9.38 – 20.81 | 0.303^d^ |
| Visual/subjective measurements | | | | | | | | | | | | |
| Flattening IVS | negative cases: 100% |  |  | negative cases: 100% |  |  | negative cases: 100% |  |  | negative cases: 100% |  | 0.624^e^ |
| RVH | negative cases: 74,4 %  positive cases: 25.6 % |  |  | negative cases: 73.2 %  positive cases: 26.8 % |  |  | negative cases: 61.1 %  positive cases: 38.9 % |  |  | negative cases: 64.7 %  positive cases: 35.3 % |  | 0.798^e^ |
| PR presence | negative cases: 28.0 %  positive cases: 72.0 % |  |  | negative cases: 20.0 %  positive cases: 80.0 % |  |  | negative cases: 22.2 %  positive cases: 77.8 % |  |  | negative cases: 25.0 %  positive cases: 75.0 % |  | 0.782^e^ |
| RA enlargement | negative cases: 74.4 %  positive cases: 25.6 % |  |  | negative cases: 77.5 %  positive cases: 22.5 % |  |  | negative cases: 64.7 %  positive cases: 35.3 % |  |  | negative cases: 76.5 %  positive cases: 23.5 % |  | 0.730^e^ |
| TR presence | negative cases: 84.7 %  positive cases: 15.3 % |  |  | negative cases: 82.9 %  positive cases: 17.1 % |  |  | negative cases: 83.3 %  positive cases: 16.7 % |  |  | negative cases: 94.1 %  positive cases: 5.9 % |  | 0.624^e^ |

^a^ median; ^b^ 25th and 75th percentile; ^c^ T-test; ^d^ Mann-Whitney U-test; ^e^ Chi-square of Pearson;

Ao, aortic annulus diameter; IVS, interventricular septum; LVIDd, left ventricular internal diameter in diastole; LVIDs, left ventricular internal diameter in systole; PA, pulmonary artery annulus diameter; PAD, pulmonary artery distensibility index; PAdia, pulmonary artery diameter in diastole; PAsys, pulmonary artery diameter in systole; PA AT, pulmonary artery acceleration time; PA AT/ET, pulmonary artery acceleration-to-ejection time ratio; PA ET, pulmonary artery ejection time; PA/Ao, pulmonary artery annulus-to-aortic annulus ratio; PR, pulmonic regurgitation; RA, right atrium; RAA, right atrium area; RVH, right ventricular hypertrophy (eccentric or concentric); TR, tricuspid regurgitation.

* The categorization was as follows: healthy (CALIF < 5 without consolidation on TUS), upper respiratory tract infection (CALIF ≥ 5 without consolidation on TUS), subclinical BP (CALIF < 5 with lobar BP), and clinical BP (CALIF ≥ 5 with lobar BP
